# Chronic Bronchitis—Spurious Melanosis of Lungs in a Dog

**Published:** 1882-10

**Authors:** 


### II.—CHRONIC BRONCHITIS—SPURIOUS MELANOSIS OF LUNGS IN A DOG.

An old Scotch terrier was sent to the Laboratory for experimental purposes, and was noticed to have a very severe cough, paroxysmal in character, much worse at some periods than at others. He was kept for several weeks, appeared in good health, took food fairly well, was not emaciated, and nothing especial was noticed in reference to the expectoration. The animal was finally killed for the purpose of examining his lungs, as it was expected that there might be something of interest from the chronic nature of the cough.

*Autopsy.*—The pathological condition was found to be confined to the lungs. The organs were crepitant throughout, and presented an unusually dark color, owing to the deposition of carbon grains in the substance. This was especially abundant immediately beneath the pleura, particularly upon the outer surface of the organ; between the fissure of the lungs the surfaces were not nearly so much pigmented.

Immediately along the sharp margins of the lobe were uniform dark lines, apparently representing the margins of larger sub-pleural lymphatics. In one or two spots this was perfectly evident; in other places the appearance of the deposit was less marked. On section of lung substance a similar pigmented condition existed, chiefly along the lines of interlobular connective tissue, but was not so evident as beneath the pleura. The mucous membrane of trachea was injected, and about the bifurcation there was distinct thickening, and the mucous membrane was rough and granular. The elastic striæ of the larger bronchi were unusually distinct; the mucosa was injected, but smooth. The tubes contained mucous. There were no parasites, no spots of consolidation of lung. The bronchial glands were carbonized, but not enlarged. On miscroscopic examination, the pigment was seen to consist, in great part, of minute irregular granules, either free in the interstices or enclosed in cells. Here and there a definite coarse particle of carbon (coal-dust) might be seen in the tissue. In thin sections, showing the pleural surface, the pigment was seen aggregated in certain definite lines representing the margin of the lymph-vessels. These aggregations were made up of closely-packed corpuscles, impregnated with carbon, some of which also existed in the form of fine granules.

*Remarks.*—The animal was an old dog, and though no history could be obtained of unusual exposure to dusty or carbonizing influences, we may assume that these had existed, as the lungs were much darker than the age of the animal would account for. So far as we can ascertain, this condition is not very common among the lower animals; though, of course, those employed in mines and mining districts suffer like the miners from the inhalation of dust, etc. In this instance, the affection of the lungs was not extensive enough to be termed anthracosis. The pigmentation may not have had anything to do with the chronic bronchitis. The rough, thickened condition of the mucosa at the bifurcation of the trachea is of interest in connection with the paroxysmal charaeter of the cough, so often met with in affections of or pressure upon this part.

